# Podocyte Injury Caused by Indoxyl Sulfate, a Uremic Toxin and Aryl-Hydrocarbon Receptor Ligand

**DOI:** 10.1371/journal.pone.0108448

**Published:** 2014-09-22

**Authors:** Osamu Ichii, Saori Otsuka-Kanazawa, Teppei Nakamura, Masaaki Ueno, Yasuhiro Kon, Weiping Chen, Avi Z. Rosenberg, Jeffrey B. Kopp

**Affiliations:** 1 Laboratory of Anatomy, Department of Biomedical Sciences, Graduate School of Veterinary Medicine, Hokkaido University, Hokkaido, Japan; 2 Kidney Disease Section, National Institute of Diabetes and Digestive and Kidney Diseases, National Institutes of Health, Bethesda, Maryland, United States of America; 3 Section of Biological Safety Research, Chitose Laboratory, Japan Food Research Laboratories, Chitose, Japan; 4 Microarray Core Facility, National Institute of Diabetes, Digestive and Kidney Diseases, National Institutes of Health, Bethesda, Maryland, United States of America; 5 Laboratory of Pathology, National Cancer Institute, National Institutes of Health, Bethesda, Maryland, United States of America; Fondazione IRCCS Ospedale Maggiore Policlinico & Fondazione D'Amico per la Ricerca sulle Malattie Renali, Italy

## Abstract

Indoxyl sulfate is a uremic toxin and a ligand of the aryl-hydrocarbon receptor (AhR), a transcriptional regulator. Elevated serum indoxyl sulfate levels may contribute to progressive kidney disease and associated vascular disease. We asked whether indoxyl sulfate injures podocytes *in vivo* and *in vitro*. Mice exposed to indoxyl sulfate for 8 w exhibited prominent tubulointerstitial lesions with vascular damage. Indoxyl sulfate-exposed mice with microalbuminuria showed ischemic changes, while more severely affected mice showed increased mesangial matrix, segmental solidification, and mesangiolysis. In normal mouse kidneys, AhR was predominantly localized to the podocyte nuclei. In mice exposed to indoxyl sulfate for 2 h, isolated glomeruli manifested increased *Cyp1a1* expression, indicating AhR activation. After 8 w of indoxyl sulfate, podocytes showed foot process effacement, cytoplasmic vacuoles, and a focal granular and wrinkled pattern of podocin and synaptopodin expression. Furthermore, vimentin and AhR expression in the glomerulus was increased in the indoxyl sulfate-exposed glomeruli compared to controls. Glomerular expression of characteristic podocyte mRNAs was decreased, including *Actn4*, *Cd2ap*, *Myh9*, *Nphs1*, *Nphs2*, *Podxl*, *Synpo*, and *Wt1*. *In vitro*, immortalized-mouse podocytes exhibited AhR nuclear translocation beginning 30 min after 1 mM indoxyl sulfate exposure, and there was increased phospho-Rac1/Cdc42 at 2 h. After exposure to indoxyl sulfate for 24 h, mouse podocytes exhibited a pro-inflammatory phenotype, perturbed actin cytoskeleton, decreased expression of podocyte-specific genes, and decreased cell viability. In immortalized human podocytes, indoxyl sulfate treatment caused cell injury, decreased mRNA expression of podocyte-specific proteins, as well as integrins, collagens, cytoskeletal proteins, and bone morphogenetic proteins, and increased cytokine and chemokine expression. We propose that basal levels of AhR activity regulate podocyte function under normal conditions, and that increased activation of podocyte AhR by indoxyl sulfate contributes to progressive glomerular injury.

## Introduction

Numerous factors likely contribute to the progression of chronic kidney disease (CKD), including the primary disease process, the effects of systemic hypertension, proteinuria, and filtered cytokines; glomerular hypertension and hyperfiltration through remnant nephrons; and the effect of various uremic toxins, which are many [1]. Indoxyl sulfate (molecular weight 212 Da), is a tryptophan metabolite that is present in plasma, where it is largely protein bound, with ∼10% being free [1]. Indoxyl sulfate is of particular interest as it has been implicated in both CKD progression and a range of manifestations of CKD, including cardiovascular disease, endothelial dysfunction, bone disease, and genomic damage [2]–[5]. At the molecular level, increased indoxyl sulfate levels are associated with oxidative stress in vascular cells, mesangial cells, and tubular epithelial cells; amplified NFκB signaling in endothelial cells and reduced Klotho expression and premature senescence of tubular epithelial cells [6]–[10]. Administration of indoxyl sulfate accelerates CKD progression in 5/6 nephrectomized rats [11], while administration of the indoxyl sulfate-binding, oral absorbent AST-120 slows the progression of glomerulosclerosis in 3/4 nephrectomized rats [12].

Total serum indoxyl sulfate levels are ∼2 µM (∼0.5 mg/L) in healthy controls and average approximately 150 µM (30 mg/L) in uremia, including CKD stage 5 and dialysis [1], [13]. Rat studies suggested that elevated serum indoxyl sulfate exacerbates glomerulosclerosis and tubulointerstitial lesions [11], [12], [14]. Indoxyl sulfate localizes to tubular cells, into which it is transported by organic anion transporters, and podocytes in uremic rats [15].

Recent studies have identified indoxyl sulfate as an endogenous ligand of the aryl hydrocarbon receptor (AhR) *in vitro* [16]. AhR acts as a ligand-activated transcription factor that regulates detoxification, carcinogenesis, and inflammation by binding to the xenobiotic response element (XRE) [17]. Other AhR ligands include exogenous environmental pollutants such as tetrachlorodibenzo-[p]-dioxin (TCDD), benzo(a)pyrene, 3-methylcholanthrene, and a range of endogenous substances including eicosanoids, bilirubin, and heme metabolites [18]. In the developing kidneys of experimental animals, TCDD causes hydronephrosis [19], [20] and reduces nephrogenesis [21]. Furthermore, cytochrome P450 1A (CYP1A), which is a highly sensitive reporter gene for AhR activation, is induced in the glomeruli of 3-methylcholanthrene-exposed mice [22], [23].

Podocytes are terminally differentiated cells that contribute to the glomerular filtration barrier [24]–[26]. Mouse podocytes express AhR, and AhR activation by TCDD alters WT1-splicing [21]. In this study, we hypothesized that excessive activation of podocyte AhR by endogenous ligands impairs podocyte function. We found that indoxyl sulfate exposure induced glomerular lesions in mice, decreased the expression of podocyte differentiation/functional markers, and induced a pro-inflammatory phenotype in mouse and human podocytes. These findings suggest that the uremic toxin indoxyl sulfate, acting via AhR, may contribute to the progression of glomerular injury in CKD.

## Methods

### Mouse studies

The animal care protocols were approved in advance by the NIDDK Animal Care and Use Committee (approval No. K097-KDB-08) and the Institutional Animal Care and Use Committee, which is convened at the Graduate School of Veterinary Medicine, Hokkaido University (approval No. 13-0032). We followed the NIH Guide for the Care and Use of Laboratory Animals and the Guide for the Care and Use of Laboratory Animals of Hokkaido University, Graduate School of Veterinary Medicine. For short-term exposure, indoxyl sulfate (Sigma-Aldrich, St. Louis, MO) dissolved in phosphate-buffered saline (PBS) was injected into C57BL/6 mice (800 mg/kg, i.p. given once). For chronic exposure, indoxyl sulfate in 4% dimethyl sulfoxide (DMSO)/PBS was injected daily into FVB/N mice, having a susceptibility to glomerular sclerosis compared to C57BL/6 mice [27], [28], for receiving 600 mg/kg, i.p. for 8 w. Kidneys, serum, and urine were collected, and glomeruli were isolated following a bead perfusion method [29]. Kidney tissue was either frozen at −80°C for RNA analysis and immunoblotting or fixed with 4% paraformaldehyde (PFA) or 2.5% glutaraldehyde for histopathological analysis. Urinary albumin/creatinine ratio (uACR) was measured using Albuwell and the Creatinine Companion (Exocell, Philadelphia, PA).

### Human kidney autopsy

Study of autopsy tissues was approved in advance by the NIH Office of Human Subjects Research (approval No. 5848); institutional policy waives consent to use tissues from deceased individuals in research. Normal renal tissue was obtained at autopsy and fixed in 10% buffered formalin.

### Indoxyl sulfate analysis by high performance liquid chromatography

Indoxyl sulfate levels in mice serum were measured by performing high performance liquid chromatography (HPLC) as described previously [30]. For binding competition, 200 µL of serum, to which we added 20 µL of 0.50 mM 1-naphthalenesulfonic acid (internal standard), was vortex-mixed with 250 µL of 0.24 M sodium octanoate (binding competitor). After incubation at room temperature for 5 min, we added 2 mL of cold acetone to precipitate the proteins. Following vortex-mixing and centrifugation at 4°C and 1, 860×*g* for 20 min, the supernatant was transferred to 12 mm×100 mm GL 14 glass test tubes and 2 mL of dichloromethane was added. After vortex-mixing and centrifuging at 4°C and 1, 860× *g* for 10 min, 200 µL of the upper layer was transferred to glass auto-sampler vials, which was followed by the addition of 20 µL of 1 M HCl. Then, 15 µL was injected onto the HPLC. We resolved the analytes on an Agilent 1100 (Agilent Technologies, Santa Clara, CA) by using reverse-phase liquid chromatography on a CapcellPak C18 UG120 (150 mm×4.6 mm; 5.0 µm particle size; Shiseido, Japan) at a flow rate of 0.6 mL/min. Mobile phase A was 0.2% trifluoroacetic acid in Milli-Q water and mobile phase B was 0.2% trifluoroacetic acid in acetonitrile. The analytical method consisted of an isocratic run with 92% mobile phase A for 30 min. Indoxyl sulfate was eluted at approximately 14 min, and the internal standard was eluted at approximately 26 min. Each analytical run was followed by a 10-min run washout gradient to 100% B. The column temperature was 25°C, and the auto-sampler tray temperature was 6°C. We quantified the analytes by using the analyte to standard peak area ratio on an Agilent 1100 fluorescence detector. The detector settings were λ_ex_ 280 nm/λ_em_ 390 nm for indoxyl sulfate and the internal standard. The calibrator containing indoxyl sulfate at a final concentration between 1.6 and 400.0 µM was prepared in Dulbecco's PBS (-). Two calibration curves were constructed with a linear response ranging from 1.6 to 32.0 µM (low) and 32.0 to 400.0 µM (high).

### Histopathological analysis

Paraffin kidney sections were stained with periodic acid Schiff, periodic acid methenamine silver, or Masson's trichrome. For electron microscopy, tissues were embedded in Quetol 812 (Nisshin EM, Japan). Ultrathin sections were doubly stained with uranyl acetate and lead citrate. For electron microscopy, mice under deep anesthesia were euthanized by cutting the vena cava and perfused via the heart with 2.5% GTA in 0.1 M phosphate buffer (pH 7.2). Isolated kidneys were then fixed with GTA and post-fixed in 1% OsO_4_.

### Immunostaining

Antigen retrieval, primary antibody, and secondary antibody for sections are described in Table S2. For immunohistochemistry, positive signals were visualized by 3,3′-diaminobenzidine. Cultured cells were washed with PBS, fixed using 4% PFA, and permeabilized with 0.3% Triton-X. Primary antibody incubation was performed at 4°C overnight (Table S1). After washing, the appropriate IgG antibody (Life Technologies, Carlsbad, CA) was reacted at room temperature for 30 min, and nuclei were stained with Hoechst dye. Fluorescence-conjugated phalloidin (Life Technologies, Carlsbad, CA) was used to label actin fibers.

### Immunoblotting

From kidneys lacking visible atrophy, soluble proteins and cytoplasmic and nuclear proteins were extracted using RIPA lysis buffer (Santa Cruz Biotechnology, Dallas, TX) or NXTREACT (Sigma-Aldrich, St. Louis, MO), respectively. Lithium dodecyl sulfate-sample buffer and sample reducing reagent (Life Technologies) were added to the samples, which were heated at 70°C for 10 min. Electrophoresis was performed on 4–12% Bis-Tris gels (Life Technologies), proteins were transferred to nitrocellulose membranes, and blocking was performed in 5% non-fat dry milk/PBS containing 0.1% Tween 20 (PBST) at room temperature for 1 h. The primary antibody was applied at 4°C overnight (Table S1). After washing with PBST, the appropriate Alexa Fluor conjugated IgG antibody (Life Technologies) was reacted at room temperature for 1 h. The intensity of each band was quantified using Image J (http://rsb.info.nih.gov/ij/), normalized against the expression of β-actin, and expressed as a ratio relative to the control group.

### RNA analysis

Total RNA was isolated from kidneys lacking visible atrophy and from cultured cells, treated with DNase, and reverse-transcribed. PCR reactions were performed with Taq polymerase (Qiagen, Venlo, Netherlands) and specific primers (Table S2). Quantitative PCR analysis was performed using SYBR Master Mix (Applied Biosystems, Carlsbad, CA). Non-template controls were included for each primer pair to assess specificity. The expression data were normalized to the expression of a housekeeping gene such as *Actb* or 18s rRNA.

### Cell culture

Mouse podocytes immortalized by temperature-sensitive SV40 large T-antigen (tsSV40) [31] and human podocytes immortalized with tsSV40 and human telomerase [32] were differentiated as described. Indoxyl sulfate (0–1.0 mM) dissolved in DMSO was added to the complete medium at day 7 (final concentration 0.1%). Cell viability was measured by CellTiter 96 non-radioactive cell proliferation assay (Promega, Fitchburg, WI). After stimulation by 0.1% DMSO or indoxyl sulfate, the cells were collected for immunoblotting or fixed using 4% PFA for immunofluorescence (Table S1). The number of Hoechst33342-positive nuclei per area and cell size were automatically counted and measured by performing fluorescence microscopy (KEYENCE, Osaka, Japan).

### Microarray analysis

Differentiated human podocytes (day 7) were stimulated with 1 mM indoxyl sulfate or 0.1% DMSO for 24 h. After stimulation, total RNA was extracted. Gene expression was analyzed using a GeneChip Human Gene 1.0 ST Array (Affymetrix, CA, USA). Microarray signals were normalized using the RMA algorithm. The significantly expressed genes were selected based on ANOVA analysis by Partek Genomics Suite (Partek, St. Charles, MO, USA). The ANOVA gene list was obtained by commercial software Partek genomic Suite. This Minimum Information About a Microarray Experiment-compliant dataset has been deposited in the NCBI Gene Expression Omnibus, GEO Series accession number GSE51834 (http://www.ncbi.nlm.nih.gov/geo/query/acc.cgi?token=ezanqyoclzunxcf&acc=GSE51834).

### Statistical analyses

The results were expressed as mean ± SD. Data for two groups were analyzed using the Student's *t-*test. For multiple comparisons, analysis was by ANOVA by using a Bonferroni test (to compare all pairs) or Dunnett test (to compare all samples vs. the control samples). Significance was inferred for *P*<0.05.

## Results

### Chronic indoxyl sulfate exposure caused renal microvascular injury in mice

**Figure 1:**
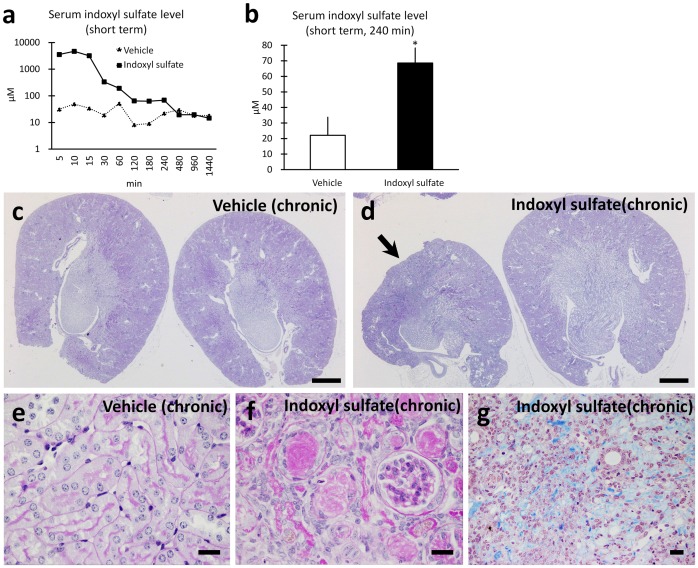
Indoxyl sulfate induced tubulointerstitial and vascular injuries in mouse kidneys. Serum indoxyl sulfate levels were measured following single dose exposure (**a** and **b**). C57BL/6 mice (800 mg/kg, i.p. given once). n = 1 (time course, panel **a**). n≥7 (240 min, panel **b**), mean ± SD. Histopathology of FVB/N mouse kidneys following chronic exposure to vehicle or indoxyl sulfate, administered at 600 mg/kg/d i.p. for 8 w (**c**–**g**). In contrast to kidneys from vehicle-exposed mice (**c**), global renal atrophy was observed in 1 of 2 kidneys from an indoxyl sulfate-exposed mouse (**d**, arrow). Bars = 500 µm. Vehicle-treated mice manifested histologically unremarkable tubules stained with periodic acid Schiff (**e**). In the macroscopically atrophied kidneys in indoxyl sulfate-exposed mice, prominent tubulointerstitial injury with numerous, prominent protein casts within tubules and extensive tubular atrophy (**f**) and foci of interstitial fibrosis were observed with Masson trichrome staining (**g**). Bars = 20 µm.

Following a single dose of indoxyl sulfate administered to mice via intraperitoneal injection, plasma levels peaked at 10 min (Figure 1a) and remained higher in indoxyl sulfate-exposed C57BL/6 mice (68 µM) compared to controls (22 µM) assessed at 240 min after dosing (Figure 1b).

For chronic exposure, we used FVB/N mice, which are more susceptible to glomerular damage compared to C57BL/6 mice [27], [28]. Indoxyl sulfate was administered by intra-peritoneal injection; a large dose is required to provide even transiently elevated plasma levels, as with normal renal function, indoxyl sulfate is rapidly excreted by glomerular filtration and tubular secretion. It is also notable that normal mice have somewhat higher plasma indoxyl sulfate levels compared to healthy humans [33].

We analyzed 7 mice following 8 w of exposure to indoxyl sulfate (Figure 1c–g). In 3 of 14 kidneys, there was severe macroscopic cortical atrophy (Figure 1d). In the macroscopically atrophic kidneys, prominent protein casts, tubular atrophy, and extensive tubular injury with tubular epithelial simplification were observed (Figure 1f). Focally, areas with a mild mononuclear infiltrate were seen in association with interstitial fibrosis (Figure 1f and g). In summary, the histological features secondary to indoxyl sulfate exposure include renal microvascular injury.

### Chronic indoxyl sulfate exposure caused glomerular damage in mice

**Figure 2:**
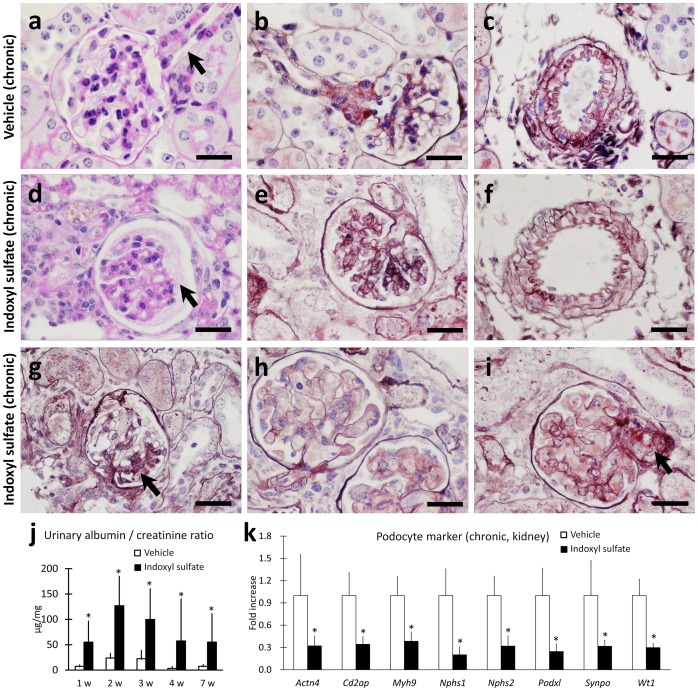
Indoxyl sulfate induced glomerular and microvascular injuries in mouse kidneys. Shown are representative images from FVB/N mice exposed to vehicle (**a**–**c**) or indoxyl sulfate (600 mg/kg, i.p. for 8 w), (**d–i**). In comparison to histologically unremarkable glomeruli (**a**), arterioles (**a**, arrow, and **b**) and arteries (**c**) in vehicle-exposed mice, glomeruli in indoxyl sulfate-exposed mice showed ischemic changes (**d** and **e**) and protein exudate in Bowman's space (**d**, arrow). In the more severely injured kidneys, glomeruli with increased mesangial matrix/segmental solidification were noted (**g**, arrow). In the kidneys with more severe injury, occasional glomeruli with mesangiolytic features were present (**h** and **i**). Histologically unremarkable mid-sized artery in vehicle-exposed mice, reduplication of elastic lamina mid-sized artery in indoxyl sulfate-exposed mouse are shown (**f**). Occasional glomerular arterioles demonstrated arteriosclerosis (**i**, arrow). Bars = 20 µm. The urinary albumin/creatinine ratio (n≥7, mean ± SD) is shown in (**j**); W denotes weeks after dosing. Podocyte marker mRNA expression in mouse kidneys is expressed as a fold change compared to the vehicle control (**k**); n≥3, mean ± S.D. * denotes significant differences between the vehicle and indoxyl sulfate groups in the same experiment (*P*<0.05).

Chronic exposure of FVB/N mice to indoxyl sulfate for 8 w produced a spectrum of glomerular and vascular injuries (Figure 2). Glomerular basement membranes showed ischemic changes with wrinkling and irregularity (GBM) (Figure 2d and e). Occasional glomeruli manifested with segmental scars (Figure 2g) and/or mesangiolytic features (Figure 2h and i). Some arterioles had constricted lumina occluded by prominent endothelial cells (Figure 2i), and some larger arteries exhibited mild reduplication of elastic lamina (Figure 2f). Furthermore, uACR significantly increased beginning 1 w after the start of indoxyl sulfate exposure and reached a peak at 2 w (Figure 2j), and the RNA expression levels of podocyte proteins in the mouse kidneys were decreased following 8 w of indoxyl sulfate exposure (Figure 2k). These results indicated glomerular damages in indoxyl sulfate-exposed mice.

### AhR localized to podocyte nuclei in mouse kidneys

**Figure 3:**
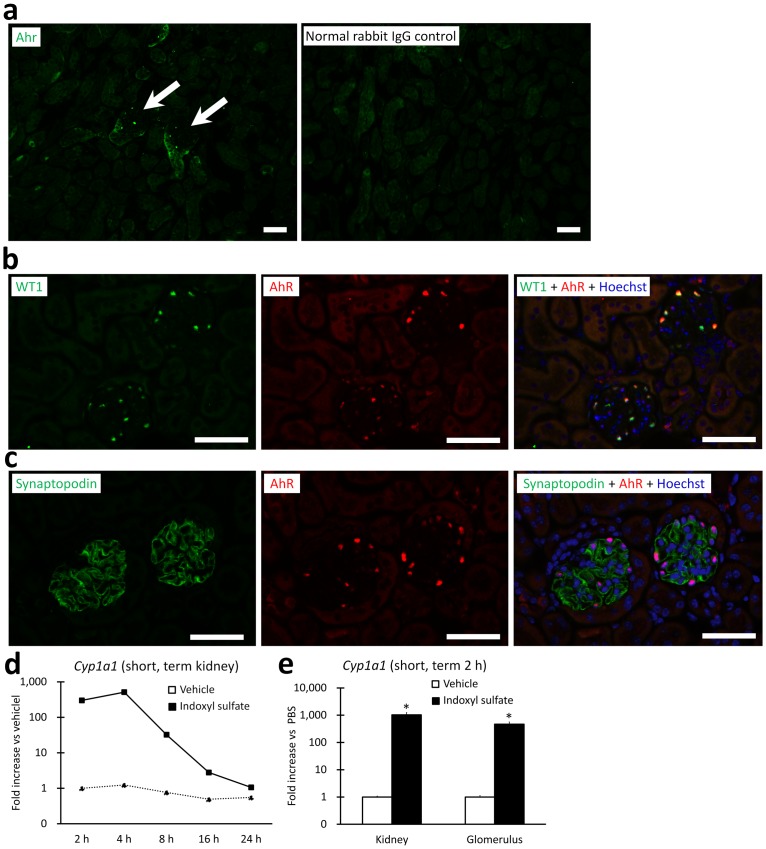
AhR localized predominantly to podocyte nuclei in mouse kidneys. Immunostaining of renal cortices from normal C57BL/6 mice for AhR (green) and normal rabbit IgG control (**a**). The arrows indicate glomeruli containing AhR-positive cells. In the renal cortex, AhR-positive cells were restricted to the glomeruli, and no positive reaction was observed in normal rabbit IgG controls. Immunostaining for WT1 (green), AhR (red), and a merged image with Hoechst nuclear stain (blue); some nuclei are yellow, suggesting that podocyte nuclei contain AhR (**b**). Immunostaining for synaptopodin (green), AhR (red), and a merged image with Hoechst nuclear stain (blue) (**c**). Nuclei expressing AhR and surrounded by synaptopodin-positive cytoplasm, confirming podocyte localization. Bars = 40 µm. The induction of *Cyp1a1* mRNA in the kidneys and glomeruli of C57BL/6 mice exposed to vehicle or indoxyl sulfate (800 mg/kg, i.p. given as a single dose). The time course of *Cyp1a1* mRNA expression in the kidneys at the time points shown following the final dose as assessed by performing real-time PCR (**d**). *Cyp1a1* mRNA expression in the kidneys and isolated glomeruli at 2 h after dose (**e**). n≥3, mean ± S.D. * denotes significant differences between the vehicle and indoxyl sulfate groups (*P*<0.05).

Indoxyl sulfate is as an endogenous ligand of AhR, acting as a ligand-activated transcription factor that regulates detoxification, carcinogenesis, and inflammation [16], [17]. We localized AhR in normal mouse kidneys by using immunofluorescence (Figure 3a–c). AhR expression was restricted to the glomerulus (Figure 3a) in a subset of podocytes positive for WT1 and synaptopodin, where it was largely restricted to the nucleus (Figure 3b and c). Next, we assessed the mRNA expression of *Cyp1a1*, which is induced by the activation of AhR following ligand binding. *Cyp1a1* mRNA expression in the kidney was significantly elevated by 2 h after exposure and peaked at 4 h, with a return to normal by 24 h, and was elevated in glomeruli as well as whole kidneys at 2 h following indoxyl sulfate exposure (Figure 3d and e).

### Chronic indoxyl sulfate exposure caused podocyte injury in mice

**Figure 4:**
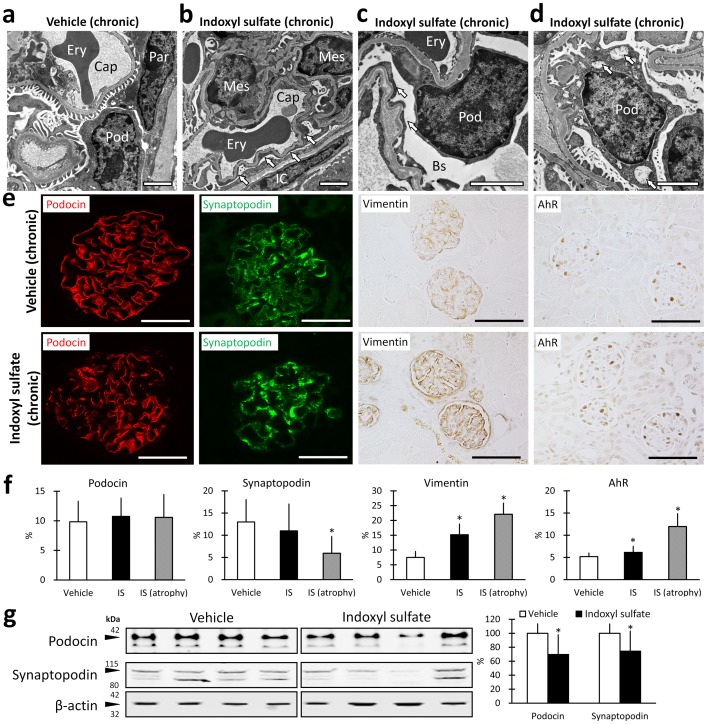
Indoxyl sulfate treatment induces podocyte injury in mice. FVB/N mice were exposed to vehicle or indoxyl sulfate (600 mg/kg, i.p.) for 8 w. Transmission electron microscopy images of glomeruli (**a–d**) demonstrate that indoxyl sulfate exposure is associated with wrinkled and partially collapsed glomerular basement (**b** and **c**) and focal podocyte (Pod) foot process effacement (**b** and **c**, arrows). Electron-lucent materials were observed in the Bowman space (Bs) in the indoxyl sulfate-exposed mouse (**c**), and the podocytes contained cytoplasmic vacuoles, consistent with protein resorption droplets (**d**, arrows). Ery denotes erythrocyte; Cap denotes capillary lumen; Par denotes parietal cell; and Mes denotes mesangial cell. Bars = 1 µm. Representative images of immunostaining of renal cortices are shown (**e**), bars = 20 µm. Histomorphometry of immune-positive glomerular area fraction that stained for podocin, synaptopodin, vimentin, and AhR in glomeruli are shown (**e**, **f**). n≥3, mean ± S.D. * denotes significant differences between the vehicle and indoxyl sulfate groups (*P*<0.05). A representative immunoblot for podocin, synaptopodin, and β-actin by using whole kidney lysate from kidneys lacking visible atrophy is shown (**f**); arrowheads indicate the predicted sizes of podocin (42 kDa), synaptopodin (100 kDa), and β-actin (42 kDa). The band intensities were quantified by performing image analysis; n = 7, mean ± S.D (**g**). * denotes significant differences between the vehicle and indoxyl sulfate groups (*P*<0.05).

We characterized podocyte injury in chronically indoxyl sulfate-exposed mice following 8 w of exposure to indoxyl sulfate (Figure 4a–g). Podocytes manifested prominent but incomplete foot process effacement and podocyte cytoplasmic vacuoles (Figure 4b–d) and a focal granular/wrinkled pattern of podocin and synaptopodin (Figure 4e). Furthermore, glomerular vimentin-positive signals and AhR-positive cells were increased in the indoxyl sulfate-exposed glomeruli compared to the control (Figure 4e). Quantitative assessment demonstrated a decrease in the synaptopodin-positive area in the glomeruli of mice showing macroscopic renal atrophy (Figure 4f). Further, a significant increase in the vimentin- or AhR-positive area was observed in indoxyl sulfate-exposed mice (Figure 4f). Immunoblotting demonstrated that podocin and synaptopodin protein levels were significantly reduced in the indoxyl sulfate-treated mice not showing macroscopic renal atrophy (Figure 4g).

### Indoxyl sulfate activated AhR and perturbed the actin cytoskeleton in cultured mouse podocytes

**Figure 5:**
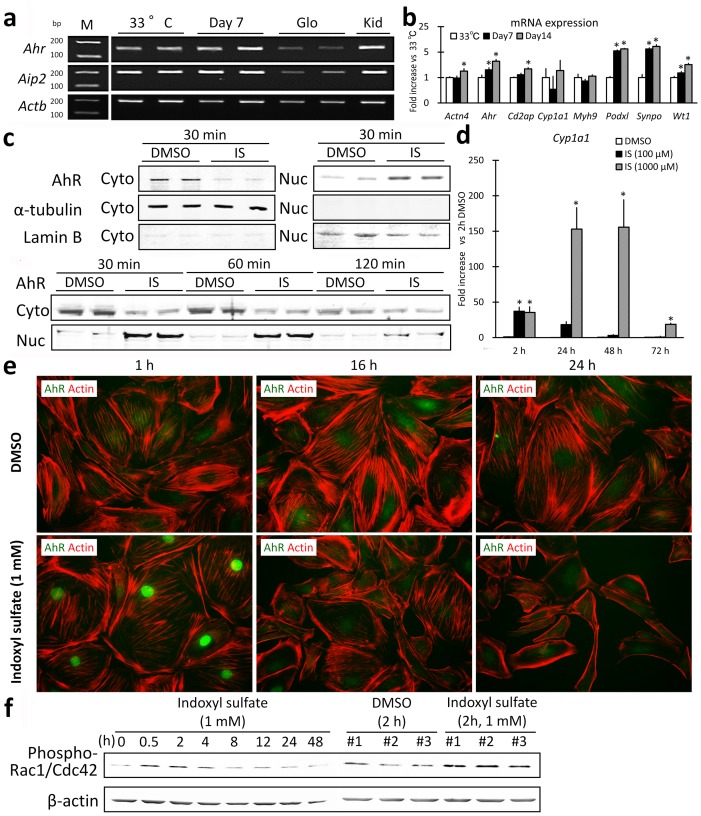
Indoxyl sulfate activated the aryl-hydrocarbon receptor and altered morphology in mouse podocytes. RT-PCR analysis for the following genes is shown: *Ahr*, *Aip*, and *Actb*, referring to aryl hydrocarbon receptor, aryl hydrocarbon receptor-interacting protein, and beta actin, respectively (**a**). M refers to the size marker. 33°C refers to mouse podocytes cultured at 33°C. Day 7 refers to mouse podocytes cultured at 37°C for 7 days. Glo and Kid denote the glomerulus and kidney isolated from an FVB/N mouse. Podocyte marker mRNA expression was measured in mouse podocytes; note that *Ahr* mRNA expression increased with podocyte differentiation (**b**). 33°C refers to mouse podocytes cultured at 33°C. Day 7 refers to mouse podocytes cultured at 37°C for 7 days. Day 14 refers to mouse podocytes cultured at 37°C for 14 days. Data generated using real-time PCR; n = 3, mean ± S.D. Fold increase vs. 33°C. * denotes significant differences vs. 33°C (*P*<0.05). Immunoblotting for AhR in differentiated mouse podocytes demonstrates nuclear translocation following indoxyl sulfate exposure (**c**). Cyto denotes cytoplasmic protein, Nuc denotes nuclear protein extracted from dimethyl sulfoxide (DMSO)-treated or indoxyl sulfate (IS)-treated mouse podocytes. α-Tubulin and Lamin B were examined to test for protein contamination with cytoplasmic or nuclear proteins, respectively. Each lane contained 20 µg of protein. In a dose-response and time-course study, *Cyp1a1* mRNA expression in indoxyl sulfate (IS)-exposed mouse podocytes was measured by real-time PCR (**d**). n = 3, mean ± S.D. Fold increase vs. each DMSO control. * denotes significant differences vs. DMSO in each time group (*P*<0.05).Immunofluorescence images of differentiated mouse podocytes exposed to DMSO or indoxyl sulfate for 1 h, 16 h, and 48 h, with staining for AhR (green) and actin (red, phalloidin staining) (**e**). Indoxyl sulfate exposure is associated with a brief, reversible migration of AhR into the nucleus. Immunoblotting for phosphorylated Rac1/Cdc42 GTPases demonstrated an increase in protein following exposure to indoxyl sulfate for 2 h (**f**). Each lane contained 5 µg of protein and triplicate wells are shown at 2 h.

mRNA expression of AhR and its partner AhR-interacting protein 2 (Aip2) was detected in mouse glomeruli and kidneys as well as cultured mouse podocytes, and the AhR mRNA expression level significantly increased with podocyte differentiation (Figure 5a and b). Following exposure to 1 mM indoxyl sulfate, nuclear translocation of AhR was clearly observed at 30 min by using proteins extracted from nuclei and the cytoplasm, and nuclear AhR was still observed at 60 min by immunoblotting (Figure 5c). *Cyp1a1* mRNA expression was induced beginning 2 h after indoxyl sulfate exposure, and this increase was sustained for 24 h with 0.1 mM indoxyl sulfate and for 72 h with 1.0 mM indoxyl sulfate (Figure 5d).

Using immunofluorescence staining (Figure 5e), we observed scant AhR protein in the nucleus or in perinuclear areas of DMSO control podocytes. Following 1.0 mM indoxyl sulfate exposure, distinct and intense AhR staining was observed in the nucleus at 60 min and was no longer present at 16 h despite continued exposure. Indoxyl sulfate exposure caused morphological changes in the mouse podocytes, inducing a more fusiform shape and reorganization of the actin cytoskeleton from stress fibers to predominantly cortical actin. Ser71 phosphorylation of Rac1/Cdc42 GTPase increases filopodial structures, cell motility, and migration [34], and phospho-Rac1/Cdc42 (Ser71) antibody detects endogenous Rac1/Cdc42 only when phosphorylated at Ser71. Phosphorylated Rac1/Cdc42 increased at 30 and 120 min after indoxyl sulfate exposure, consistent with a shift toward a motile phenotype (Figure 5f).

### Indoxyl sulfate altered the expression of differentiation markers in mouse podocytes *in vitro*

**Figure 6:**
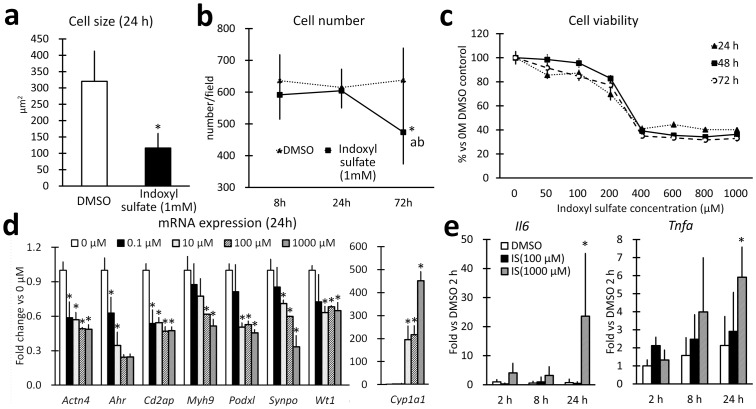
Indoxyl sulfate altered differentiation marker expression in mouse podocytes. The size of differentiated mouse podocytes decreased with indoxyl sulfate compared to dimethyl sulfoxide (DMSO) control; n = 3, mean ± SD (**a**). * denotes significant differences between the DMSO and indoxyl sulfate groups (*P*<0.05). Cell numbers were reduced in indoxyl sulfate-treated mouse podocytes compared to those treated with DMSO; n = 3, mean ± SD (**b**). Indoxyl sulfate-treated cells were reduced in number at 72 h compared to DMSO control (*, *P*<0.05). Indoxyl sulfate-treated cells were reduced at 72 h compared to the 8 h (a, *P*<0.05) and 24 h (b, P<0.05) time points. A dose-response study showed that the viability of differentiated podocytes, assessed using an MTT assay, was reduced to a similar extent at 24, 48, and 72 h, and that the toxic effect reached a plateau at 400 µM; n = 3, mean ± SD (**c**). The baseline viability was assessed using a 0-µM control for each time group. Podocyte marker mRNA expression was reduced by indoxyl sulfate, as assessed by real-time PCR in differentiated mouse podocytes after indoxyl sulfate treatment (**d**); n = 3, mean ± S.D. Data are presented as fold increase vs. DMSO (0 µM). * denotes significant differences vs. control for each gene (*P*<0.05). RNA expression of two cytokines, *Il6* and *Tnfa*, increased in differentiated mouse podocytes after indoxyl sulfate (IS) treatment (**e**); n = 3, mean ± S.D, fold increase vs. DMSO in each gene. * denotes significant differences vs. DMSO for each time group (*P*<0.05); h denotes hours after exposure.

In mouse podocytes exposed to 1 mM indoxyl sulfate, cell size and number decreased, and cell viability decreased in a dose-dependent manner (Figure 6a–c). The expression of podocyte markers was significantly decreased by 24 h indoxyl sulfate exposure in a dose-dependent fashion, and *Ahr* was downregulated (Figure 6d). Remarkably, mRNA expression of inflammatory factors that are associated with glomerular injuries, [35]–[37] such as *Il6* and *Tnfa*, was significantly induced following indoxyl sulfate exposure (Figure 6e).

### Indoxyl sulfate altered the morphology and decreased the viability of human podocytes *in vitro*

**Figure 7:**
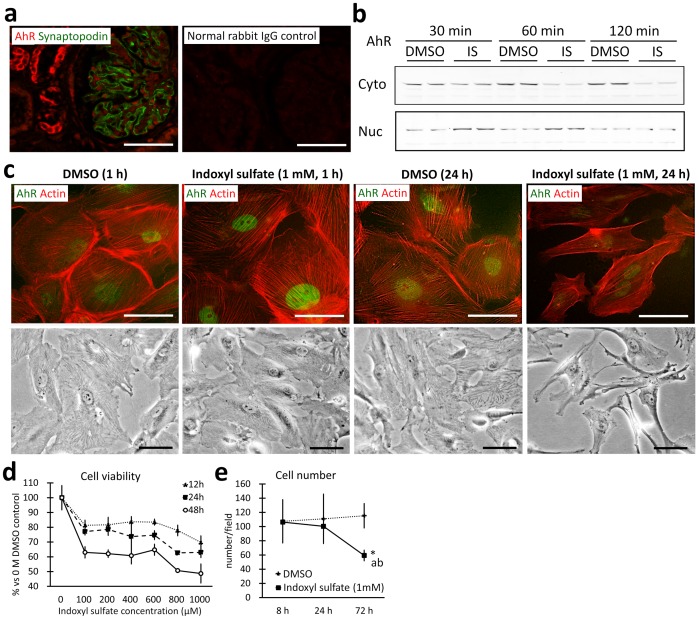
Indoxyl sulfate injures human podocytes. Immunofluorescence images of autopsied human kidneys shows juxtaposition of AhR in podocyte nuclei surrounded by cytoplasm expressing synaptopodin (**a**). AhR (red), synaptopodin (green), and normal rabbit IgG control. Immunoblotting for AhR in differentiated human podocytes demonstrates nuclear translocation following indoxyl sulfate exposure (**b**). Cyto denotes cytoplasmic protein, Nuc denotes nuclear protein extracted from dimethyl sulfoxide (DMSO)- or indoxyl sulfate (IS)-treated human podocytes. Each lane contained 20 µg of protein. Immunofluorescence and phase-contrast images of differentiated human podocytes exposed to DMSO or indoxyl sulfate for 1 h shows rapid nuclear localization following indoxyl sulfate exposure (**c**). AhR (green). Actin (red, phalloidin staining). Cell viability, assessed using an MTT assay, of indoxyl sulfate-treated differentiated human podocytes was reduced in a dose-dependent and time-dependent fashion compared to DMSO control (**d**). n = 3, mean ± SD. Baseline viability was assessed using a 0 µM control for each time group. Cell numbers were deceased in indoxyl sulfate-treated differentiated human podocytes compared to DMSO control (**e**). n = 3, mean ± S.D. Indoxyl sulfate-treated cells were reduced in number at 72 h compared to DMSO control (*, *P*<0.05). Indoxyl sulfate-treated cell numbers were also reduced at 72 h compared to the 8 h (a, *P*<0.05) and 24 h (b, *P*<0.05) time points.

In normal human kidneys obtained at autopsy, AhR was localized to the distal tubule cytoplasm, where a particularly strong signal was detected, and podocyte nuclei (Figure 7a). In cultured immortalized human podocytes, 1 mM indoxyl sulfate exposure caused AhR nuclear translocation beginning at 30 min, decreased cell size and actin fibers, and shifted cell shape from polygonal to fusiform at 24 h (Figure 7b and c). Cell numbers decreased in a time- and dose-dependent fashion, while cell viability decreased over time (Figure 7d and e).

### Indoxyl sulfate downregulated differentiation markers and upregulated inflammatory mediators in human podocytes

We performed comparative microarray analysis, comparing DMSO- and indoxyl sulfate-exposed human podocytes (1 mM for 24 h), focusing on genes related to podocyte injury, function, and inflammation. Among the selected podocyte-specific mRNAs, *SYNPO*, *ACTN4*, *CDH13*, *MME*, *VIM*, *DAG1*, *FAT1*, and *CDH3* [25], [26], [38] were significantly downregulated in indoxyl sulfate-exposed podocytes (Table 1). Indoxyl sulfate exposure also decreased the expression of collagens that mediate capillary morphology and glomerular function [24], integrins that mediate cell adhesion [25], [26], [39], [40], myosin/actin that constitute the cytoskeleton, and bone morphogenetic proteins that mediate kidney development and repair [41]. Further, inflammatory molecules were significantly elevated in the indoxyl sulfate-exposed podocytes (Table 2).

**Table 1:** Altered expression of podocyte function-associated genes in indoxyl sulfate-exposed human podocytes.

Rank	Gene symbol	Gene name	Refseq		*P*-value		Fold change
**Podocyte functions**							
1	*MAP1LC3B*	microtubule-associated protein 1 light chain 3 beta	NM_022818		0.001	*	1.36
2	*PDPN*	podoplanin	NM_006474		0.003	*	1.28
3	*MAP1LC3B2*	microtubule-associated protein 1 light chain 3 beta 2	NM_001085481		0.000	*	1.22
4	*WT1*	Wilms tumor 1	NM_024424		0.111		1.20
5	*FAT2*	FAT tumor suppressor homolog 2 (*Drosophila*)	NM_001447		0.359		1.13
6	*CD2AP*	CD2-associated protein	NM_012120		0.070		1.09
7	*KIRREL*	kin of IRRE-like (*Drosophila*)	NM_018240		0.159		1.08
8	*DES*	desmin	NM_001927		0.568		1.07
9	*PTPRO*	protein tyrosine phosphatase, receptor type, O	NM_030667		0.514		1.04
10	*MAP1LC3C*	microtubule-associated protein 1 light chain 3 gamma	NM_001004343		0.370		1.03
11	*LMX1B*	LIM homeobox transcription factor 1, beta	NM_002316		0.487		0.98
12	*TJP1*	tight junction protein 1 (zona occludens 1)	NM_003257		0.177		0.97
13	*MYOC*	myocilin, trabecular meshwork inducible glucocorticoid response	NM_000261		0.755		0.96
14	*NPHS1*	nephrosis 1, congenital, Finnish type (nephrin)	NM_004646		0.274		0.93
15	*NPHS2*	nephrosis 2, idiopathic, steroid-resistant (podocin)	NM_014625		0.181		0.93
16	*MAP1LC3A*	microtubule-associated protein 1 light chain 3 alpha	NM_032514		0.514		0.92
17	*EZR*	ezrin	NM_003379		0.068		0.92
18	*CD80*	CD80 molecule	NM_005191		0.377		0.91
19	*SYNPO2*	synaptopodin 2	NM_133477		0.484		0.91
20	*PODXL*	podocalyxin-like	NM_001018111		0.215		0.90
21	*CDH3*	cadherin 3, type 1, P-cadherin (placental)	NM_001793		0.040	*	0.88
22	*FAT1*	FAT tumor suppressor homolog 1 (*Drosophila*)	NM_005245		0.007	*	0.87
23	*PODXL2*	podocalyxin-like 2	NM_015720		0.214		0.87
24	*DAG1*	dystroglycan 1 (dystrophin-associated glycoprotein 1)	NM_001165928		0.034	*	0.85
25	*VIM*	vimentin	NM_003380		0.000	*	0.85
26	*MME*	membrane metallo-endopeptidase	NM_007288		0.001	*	0.83
27	*CDH13*	cadherin 13, H-cadherin (heart)	NM_001257		0.000	*	0.72
28	*ACTN4*	actinin, alpha 4	NM_004924		0.000	*	0.67
29	*SYNPO*	synaptopodin	NM_007286		0.000	*	0.66
**Collagens**							
1	*COL4A5*	collagen, type IV, alpha 5	NM_000495		0.000		0.79
2	*COL5A2*	collagen, type V, alpha 2	NM_000393		0.024		0.78
3	*COL1A1*	collagen, type I, alpha 1	NM_000088		0.001		0.63
4	*COL4A2*	collagen, type IV, alpha 2	NM_001846		0.000		0.63
5	*COL4A1*	collagen, type IV, alpha 1	NM_001845		0.000		0.57
6	*COL8A1*	collagen, type VIII, alpha 1	NM_001850		0.000		0.56
7	*COL12A1*	collagen, type XII, alpha 1	NM_004370		0.000		0.51
8	*COL11A1*	collagen, type XI, alpha 1	NM_001854		0.000		0.50
**Integrins**							
1	*ITGB2*	integrin, beta 2	NM_000211		0.021		0.82
2	*ITGB5*	integrin, beta 5	NM_002213		0.002		0.78
3	*ITGB4*	integrin, beta 4	NM_000213		0.010		0.75
4	*ITGB3*	integrin, beta 3	NM_000212		0.000		0.66
5	*ITGB6*	integrin, beta 6	NM_000888		0.001		0.41
**Myosins and Actins**							
1	*MYH16*	myosin, heavy chain 16 pseudogene	NR_002147		0.003		1.56
2	*MYH10*	myosin, heavy chain 10, non-muscle	NM_005964		0.005		0.77
3	*MYH9*	myosin, heavy chain 9, non-muscle	NM_002473		0.001		0.73
4	*ACTA2*	actin, alpha 2, smooth muscle, aorta	NM_001141945		0.004		0.72
**Bone morphogenetic proteins**							
1	*BMP1*	bone morphogenetic protein 1	NM_006129		0.000		0.78
2	*BMP4*	bone morphogenetic protein 4	NM_001202		0.003		0.71

n = 3. Podocytes were exposed to 0.1% dimethyl sulfoxide or 1.0 mM indoxyl sulfate for 24 h. Fold change indicates the values for the indoxyl sulfate group vs. dimethyl sulfoxide control. Genes associated with podocyte function were selected (*: significant differences, *P*<0.05). Other genes (*P*<0.05 and fold change  =  1.20 ≧ or 0.83 ≦).

**Table 2:** Altered expression of inflammation-associated genes in indoxyl sulfate-exposed human podocytes.

Rank	Gene symbol	Gene name	Refseq	*P*-value	Fold change
**Interleukins and receptors**					
1	*IL1B*	interleukin 1, beta	NM_000576	0.0000	9.43
2	*IL1A*	interleukin 1, alpha	NM_000575	0.0000	3.92
3	*IL1R1*	interleukin 1 receptor, type I	NM_000877	0.0001	2.59
4	*IL1F9*	interleukin 1 family, member 9	NM_019618	0.0003	1.97
5	*IL12A*	interleukin 12A	NM_000882	0.0055	1.68
6	*IL8*	interleukin 8	NM_000584	0.0030	1.47
7	*IL6ST*	interleukin 6 signal transducer	NM_002184	0.0048	1.25
8	*IL18R1*	interleukin 18 receptor 1	NM_003855	0.0034	1.22
9	*IL6*	interleukin 6 (interferon, beta 2)	NM_000600	0.0105	1.21
10	*IL11RA*	interleukin 11 receptor, alpha	NM_147162	0.0181	0.82
11	*IL32*	interleukin 32	NM_001012631	0.0167	0.72
12	*IL18*	interleukin 18	NM_001562	0.0061	0.70
**Chemokine (C-X-C motif) ligands**					
1	*CXCL2*	chemokine (C-X-C motif) ligand 2	NM_002089	0.0005	2.37
2	*CXCL12*	chemokine (C-X-C motif) ligand 12	NM_000609	0.0099	1.60
3	*CXCL5*	chemokine (C-X-C motif) ligand 5	NM_002994	0.0004	1.50
4	*CXCL3*	chemokine (C-X-C motif) ligand 3	NM_002090	0.0023	1.46
5	*CXCL1*	chemokine (C-X-C motif) ligand 1	NM_001511	0.0005	1.42
**Chemokine (C-C motif) ligands**					
1	*CCL20*	chemokine (C-C motif) ligand 20	NM_004591	0.0000	3.24
2	*CCL3*	chemokine (C-C motif) ligand 3	NM_002983	0.0002	1.99
3	*CCL3L1*	chemokine (C-C motif) ligand 3-like 1	NM_021006	0.0003	1.86
4	*CCL2*	chemokine (C-C motif) ligand 2	NM_002982	0.0004	1.46
5	*CCL4L1*	chemokine (C-C motif) ligand 4-like 1	NM_001001435	0.0411	1.28
6	*CCL11*	chemokine (C-C motif) ligand 11	NM_002986	0.0490	0.73
7	*CCL5*	chemokine (C-C motif) ligand 5	NM_002985	0.0158	0.62
**Other inflammatory molecules**					
1	*CSF2*	colony-stimulating factor 2	NM_000758	0.0002	3.03
2	*CSF3*	colony-stimulating factor 3	NR_033662	0.0011	1.50
3	*VEGFA*	vascular endothelial growth factor A	NM_001025366	0.0009	1.48
4	*NAMPT*	nicotinamide phosphoribosyltransferase	NM_005746	0.0023	1.43
5	*TNFSF13B*	tumor necrosis factor (ligand) superfamily, member 13b	NM_006573	0.0102	1.42
6	*TNF*	tumor necrosis factor	NM_000594	0.0338	1.39
7	*C3*	complement component 3	NM_000064	0.0002	0.74
8	*MIF*	macrophage migration inhibitory factor	NM_002415	0.0329	0.63

n = 3. Podocytes were exposed to 0.1% dimethyl sulfoxide or 1.0 mM indoxyl sulfate for 24 h. Fold change indicates the values for the indoxyl sulfate group vs. dimethyl sulfoxide control. Genes (*P*<0.05 and fold change  =  1.20 ≧ or 0.83 ≦).

## Discussion

Previous studies have shown that AhRs localize to the renal and collecting tubules of human fetal kidneys [42], as well as to podocytes in fetal and adult mouse kidneys [21]. Consistent with the latter report, our results showed that AhR localized to podocyte nuclei in adult mouse and human kidneys, as well as to distal tubules in human kidneys. These data may suggest species- or age-dependent differences in AhR activation. Since AhR is a ligand-binding transcription factor [17], nuclear localization of podocyte AhR likely indicates the constitutive binding of endogenous ligands in adult kidneys. AhR endogenous ligands are believed to include eicosanoids and bilirubin [18], and indoxyl sulfate was recently reported to be an AhR ligand in liver cell lines [16]. Further, AhR in podocytes was observed in the nucleus *in vivo* but in the cytoplasm *in vitro*. Our data suggest that podocytes may also be a target of circulating endogenous AhR ligands. Interestingly, using a monoclonal indoxyl sulfate antibody [43], indoxyl sulfate has been detected in podocytes as well as tubular cells in uremic rats [15]. Therefore, we propose that increased plasma levels of endogenous ligands, such as indoxyl sulfate, activate podocyte AhR, thereby promoting a transcriptional program that drives progressive glomerular damage.

Increased serum indoxyl sulfate has been associated with tubulointerstitial damage [12], [13], [44] and indoxyl sulfate also exacerbates glomerular injury [11]. AST-120 therapy in rodent models of kidney disease, which reduce plasma indoxyl sulfate levels, slows the progression of glomerulosclerosis and tubulointerstitial damage [12], [14]. Tubulointerstitial damage in indoxyl sulfate-exposed mice may be caused by mechanisms such as oxidative stress in tubular cells [9], [10], [45], [46]. Some indoxyl sulfate-treated mice showed severe tubulointerstitial damage, and 3 out of 14 total kidneys displayed macroscopic renal cortex atrophy. As part of the supplemental data, we also analyzed the histopathology of indoxyl sulfate-treated mice after heminephrectomy (Figure S1). In this experiment, although uACR was significantly increased in indoxyl sulfate-treated mice compared with vehicle-treated controls, the histological scores of tubulointerstitial lesions did not significantly differ between the two groups, and individual differences in tubuloineterstitial lesions remained in the indoxyl sulfate-treated group. Further, macroscopic renal cortex atrophy in indoxyl sulfate-treated kidneys is likely an end-stage feature caused by the summation of glomerular and tubulointerstitial injuries [47]. Therefore, the differences in macroscopic renal cortex atrophy observed among individuals would indicate differences in pathological stage among kidneys treated with indoxyl sulfate. Thus, there would be a threshold at which indoxyl sulfate causes cell damage in mouse kidneys, and indoxyl sulfate-treated kidneys might show macroscopic renal atrophy with tubulointerstitial lesions when cell damage passed this threshold. Further, as shown in Figure 1d, the macroscopic atrophy was observed focally. This result might indicate vasculocentric pattern associating to end stage process of tubular atrophy and interstitial fibrosis. One possibility is the degree of endothelial toxicity/injury differs between the larger and smaller vessels with greater toxicity to smaller vessels in contrast to large ones. This would be most particularly the point made by showing the different form of ischemic injury likely resulting from microvascular damage. Indoxyl sulfate is derived from intestinal tryptophan, and the activation of AhR is affected by various endogenous ligands. Therefore, multiple environmental or physiological factors might also cause individual differences in macroscopic renal atrophy among indoxyl sulfate-treated kidneys.

We found that the administration of indoxyl sulfate to healthy mice produced glomerulopathy and tubulointerstitial damage. Brief exposure of mice to indoxyl sulfate increased serum indoxyl sulfate and *Cyp1a1* expression in the glomerulus, and chronic indoxyl sulfate exposure significantly increased uACR, likely reflecting glomerular dysfunction. Indoxyl sulfate-exposed mice developed wrinkled GBM, foot process effacement, and cytoplasmic vacuoles in podocytes. Taken in their entirety, our findings suggest that increased serum indoxyl sulfate induced glomerular AhR activation and podocyte injury. The decreased expression of collagens and integrins in indoxyl sulfate-exposed human podocytes *in vitro* may reflect morphological changes in indoxyl sulfate-exposed glomeruli *in vivo*. In addition, recent reports have indicated that indoxyl sulfate also mediates injury of endothelial cells and mesangial cells by production of reactive oxygen species [8], [48].

In mouse and human podocytes, indoxyl sulfate caused AhR nuclear translocation, *Cyp1a1* induction, and decreased cell size and viability. These results indicate that indoxyl sulfate functions, at least in part, as a ligand of podocyte AhR. The decreased cell number of podocytes following indoxyl sulfate exposure may reflect cell death or detachment from the culture substratum. In fact, apoptosis and altered adherens junctions have been reported in indoxyl sulfate-exposed endothelial cells and renal tubular cells [49], [50], and our microarray results for indoxyl sulfate-exposed human podocytes also showed decreased expression of integrins, which facilitate podocyte adhesion to the extracellular matrix [25], [39], [51].

Furthermore, chronically indoxyl sulfate-exposed kidneys showed decreased expression and/or altered staining patterns for podocin (slit diaphragm) and synaptopodin (actin cytoskeleton) and increased expression of vimentin (an intermediate filament protein), indicating podocyte injury [52]. Further, we have recently demonstrated that the decreased mRNA expression of podocyte markers, as observed in indoxyl sulfate-exposed mice, is closely correlated with podocyte injury in murine glomerulonephritis [53]. We propose that indoxyl sulfate and AhR activation mediate podocyte injury by decreasing the expression of critical podocyte proteins that regulate the slit diaphragm, cell morphology, and matrix adhesion. In particular, indoxyl sulfate strongly altered the expression and organization of cytoskeleton molecules such as actin in mouse and human podocytes. Interestingly, 3-methylcholanthrene, which is an AhR ligand, upregulates Rho-GTPase family RNA and proteins in endothelial cells [54]. In a previous study using podocyte-specific Cdc42, Rac1, and RhoA knockout mice, the physiological importance of Cdc42, but not Rac1 or RhoA, in podocytes was shown [55]. Further, the reorganization of the cytoskeletal network mediated by Rac1 and Cdc42 plays a primary role in the cellular responses to podocyte-specific proteins [56]. Rac1 and Cdc42 have been shown to have the opposite function to RhoA in podocytes [57]. Ser71 phosphorylation of Rac1/Cdc42 increases filopodial structures, cell motility, and migration [34]. Therefore, in the present study, an increase in phosphorylated Rac1/Cdc42 was observed after indoxyl sulfate exposure in mouse podocytes. The Rho-GTPase family regulates cytoskeletal dynamics, and a shift from RhoA activity to Rac1 activity is associated with increased podocyte motility *in vitro* and proteinuria *in vivo* [58].

Podocyte injury by indoxyl sulfate may involve additional pathways that may be downstream of AhR or independent of AhR. Indoxyl sulfate has been shown to damage tubular epithelial cells via Stat3 activation [59] and to impair mitochondrial function [60]. Furthermore, while we have presented data supporting a direct role for indoxyl sulfate in podocyte injury *in vitro*, it remains possible that podocyte injury *in vivo* is due at least in part to chronic vascular damage by indoxyl sulfate. In a preliminary experiment, we performed AhR-knockdown experiments using siRNA and immortalized-mouse podocytes, and observed that mRNA expression of podocyte functional genes decreased after AhR-knockdown (Figure S2). In order to examine this issue directly, we are currently generating mice in which AhR is deleted from podocytes.

Activated AhR translocates to the nucleus with a partner, such as ARNT, binds to XRE sequences, and regulates expression of target genes such as *Cyp1a1* [17]. Beyond the classical AhR/ARNT pathway, RelA and RelB, which are components of the NFκB protein complex, also function as AhR partners and regulate the expression of IL-6, IL-8, and chemokines [61]. This observation in part explains our results that indoxyl sulfate induced inflammatory genes in podocytes. In addition to these multiple AhR transcriptional partners, ligand-dependent function of AhR has been reported [62]. Therefore, the possibility of both podocyte-specific AhR partners and ligand-independent AhR activation in normal podocytes might explain why AhR localized to podocyte nuclei rather than other renal cells in normal mouse kidneys. From the UCSC Genome Bioinformatics database (https://genome.ucsc.edu/), we obtained the core XRE sequence (GCGTG) and searched 3000 bp upstream of the transcription initiation site of podocyte-specific genes. We identified the core XRE sequence in *Actn4* (2 sites), *Cd2ap* (5 sites), *Myh9* (3 sites), *Nphs2* (1 site), *Podxl* (2 sites), and *Synpo* (2 sites).

In conclusion, we found that podocyte AhR activated by indoxyl sulfate causes glomerular damage and podocyte injury. This damage is characterized by altered cell morphology, reduced expression of podocyte differentiation markers, and a pro-inflammatory state. Our findings suggest a novel mechanism by which the uremic toxin indoxyl sulfate may drive the progression of glomerular injury.

## Supporting Information

Figure S1 **Clinical and histological scores of indoxyl sulfate-treated mice after heminephrectomy.** FVB/N mice were heminephrectomized at 2 months old, and indoxyl sulfate (IS) was administered after mice were 3 months old. **(a)** Urinary albumin/creatinine ratio. Mean ± SD, n = 9. *: significant difference compared with vehicle control at day 30 after dose (*P*<0.05). #: significant difference compared to IS-treated mice at day 7 after dose (*P*<0.05). **(b)** Number of proteins cast in renal medulla (2 sections/1 mouse), mean ± SD. **(c)** Semi-quantitative scores of tubulointerstitial lesions (2 sections/1 mouse), mean ± SD.
(DOCX)

Figure S2 **AhR knock-down altered the expression of markers of differentiation in mouse podocytes.** To suppress the expression of target mRNA, we used pre-designed siRNA (Integrated DNA Technologies, Coralville, IA). Trypsinized mouse podocytes were cultured with complete medium without antibiotics in 24 well plates at 60–70% confluence for 2 days. At day 3, the medium was changed to a mixture of Lipofectamine 2000 and Opti-MEM (Life Technologies) containing negative control RNA (40 pM, AllStars Negative Control; Qiagen, Venlo, Netherlands) or pre-designed siRNA (40 pM, Screening DsiRNA Duplex, Integrated DNA Technologies). mRNA expression of podocyte-specific proteins after 24 h and 48 h of RNAi treatment. Real-time PCR. n = 3, mean ± S.D. Fold increase vs. oligo-treated controls. *: Significant differences vs. control for each gene (*P*<0.05).
(DOCX)

Table S1 **Antibodies used in this study.**
(DOCX)

Table S2 **Primers used in this study.**
(DOCX)
