# Supplementary figures and images for: Podocyte Injury Caused by Indoxyl Sulfate, a Uremic Toxin and Aryl-Hydrocarbon Receptor Ligand
Source: PLoS One. 2014 Sep 22;9(9):e108448. doi: 10.1371/journal.pone.0108448 (PMC4171541; doi:10.1371/journal.pone.0108448)

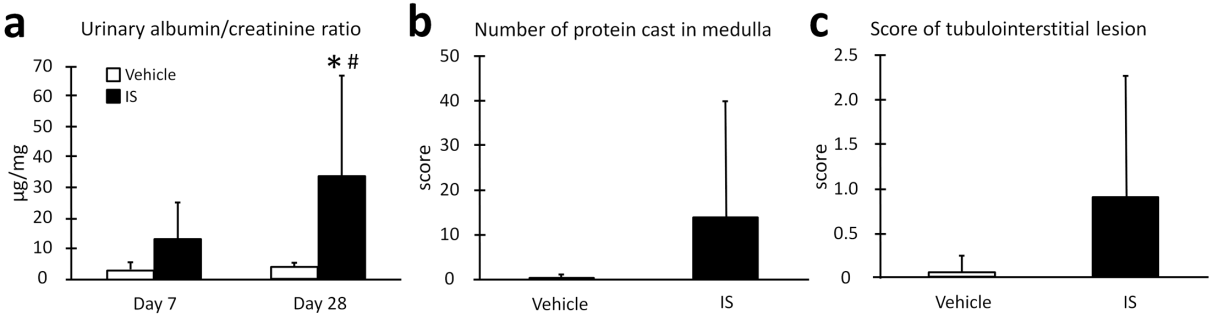

Supplement: Figure S1 — Clinical and histological scores of indoxyl sulfate-treated mice after heminephrectomy. FVB/N mice were heminephrectomized at 2 months old, and indoxyl sulfate (IS) was administered after mice were 3 months old. (a) Urinary albumin/creatinine ratio. Mean ± SD, n = 9. *: significant difference compared with vehicle control at day 30 after dose (P<0.05). #: significant difference compared to IS-treated mice at day 7 after dose (P<0.05). (b) Number of proteins cast in renal medulla (2 sections/1 mouse), mean ± SD. (c) Semi-quantitative scores of tubulointerstitial lesions (2 sections/1 mouse), mean ± SD. (DOCX) [file pone.0108448.s001.docx]

**
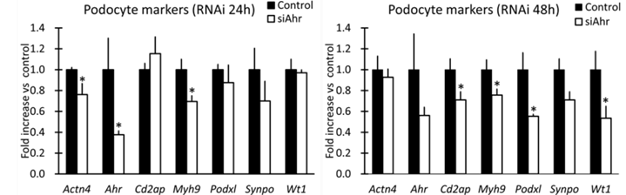
**

Supplement: Figure S2 — AhR knock-down altered the expression of markers of differentiation in mouse podocytes. To suppress the expression of target mRNA, we used pre-designed siRNA (Integrated DNA Technologies, Coralville, IA). Trypsinized mouse podocytes were cultured with complete medium without antibiotics in 24 well plates at 60–70% confluence for 2 days. At day 3, the medium was changed to a mixture of Lipofectamine 2000 and Opti-MEM (Life Technologies) containing negative control RNA (40 pM, AllStars Negative Control; Qiagen, Venlo, Netherlands) or pre-designed siRNA (40 pM, Screening DsiRNA Duplex, Integrated DNA Technologies). mRNA expression of podocyte-specific proteins after 24 h and 48 h of RNAi treatment. Real-time PCR. n = 3, mean ± S.D. Fold increase vs. oligo-treated controls. *: Significant differences vs. control for each gene (P<0.05). (DOCX) [file pone.0108448.s002.docx]
