# Supplementary material for: Podocyte Injury Caused by Indoxyl Sulfate, a Uremic Toxin and Aryl-Hydrocarbon Receptor Ligand
Source: PLoS One. 2014 Sep 22;9(9):e108448. doi: 10.1371/journal.pone.0108448 (PMC4171541; doi:10.1371/journal.pone.0108448)
Supplement: Table S1 — Antibodies used in this study. (DOCX) [file pone.0108448.s003.docx]

|  |  | **Actin-beta** |  | **Alpha-tubulin** |  | **Aryl-hydrocarbon receptor** |  | **Lamin B** |  | **Vimentin** |  | **Podocin** |  | **Phospho- Rac1/Cdc42** |  | **Synaptopodin** |  | **Wilms’ tumor 1** |  | **Incubation** |
| --- | --- | --- | --- | --- | --- | --- | --- | --- | --- | --- | --- | --- | --- | --- | --- | --- | --- | --- | --- | --- |
| **Primary antibody for IHC and IF** |  | None |  | Mouse monoclonal antibodies (No. ab7291, 1:5000; Abcam, MA, USA) |  | Rabbit polyclonal antibodies (No. SA550, 1:600; Enzo Life Sciences, Farmingdale, NY, USA) |  | Rabbit polyclonal antibodies (No. sc-6217, 1:500; Santa Cruz, Dallas, TX, USA) |  | Rabbit monoclonal antibodies (No. 5741, 1:1000; Cell Signaling, MA, USA) |  | Rabbit polyclonal antibodies (No. 29070, 1:800; IBL, Gunma, Japan) |  | None |  | Mouse monoclonal antibodies (No. 10R-S125a, 1:50; Fitzgerald, MA, USA) |  | Mouse monoclonal antibodies (No. 05-753, 1:300; Merck Millipore, Billerica, MA, USA) |  | 4°C, overnight |
| **Secondary antibody for IHC** |  | None |  | None |  | Biotinylated goat anti-rabbit IgG antibodies (SABPO kit, Nichirei) |  | None |  | Biotinylated goat anti-rabbit IgG antibodies (SABPO kit, Nichirei) |  | None |  | None |  | None |  | None |  | Room temperature, 30 min |
| **Secondary antibody for IF** |  | None |  | None |  | Alexa Fluor 488 or 546-labeled donkey anti-rabbit IgG antibodies (1:500; Life Technologies, Carlsbad, CA, USA) |  | None |  | None |  | Alexa Fluor 546-labeled donkey anti-rabbit IgG antibodies (1:500; Life Technologies, Carlsbad, CA, USA) |  | None |  | Alexa Fluor 488-labeled donkey anti-mouse IgG antibodies (1:500; Life Technologies, Carlsbad, CA, USA) |  | Alexa Fluor 488-labeled donkey anti-mouse IgG antibodies (1:500; Life Technologies, Carlsbad, CA, USA) |  | Room temperature, 30 min |
| **Antigen retrieval in section** |  | None |  | None |  | Citrate buffer (pH 6.0) |  | None |  | Citrate buffer (pH 6.0) |  | Citrate buffer (pH 6.0) |  | None |  | Dako Target Retrieval Solution at pH 9 (DAKO, Glostrup, Denmark) |  | Citrate buffer (pH 6.0) |  | Heating, 105°C, 15 min |
| **Primary antibody for WB** |  | Mouse monoclonal antibodies (No. A2228, 1:10000; Sigma-Aldrich, St. Louis, MO, USA) |  | None |  | Rabbit polyclonal antibodies (No. SA550, 1:100; Enzo Life Sciences, Farmingdale, NY, USA) |  | None |  | None |  | Rabbit polyclonal antibodies (No. 29070, 1:1000; IBL, Gunma, Japan) |  | Rabbit polyclonal antibodies (No. 2461, 1:1000; Cell Signaling Technology, Danvers, MA, USA) |  | Mouse monoclonal antibodies (No. 10R-S125a, 1:100; Fitzgerald, MA, USA) |  | None |  | 4°C, overnight |
| **Secondary antibody for WB** |  | Alexa Fluor 488-labeled donkey anti-mouse IgG antibodies (1:5000, Life Technologies, Carlsbad, CA, USA) |  | Alexa Fluor 488-labeled donkey anti-rabbit IgG antibodies (1:5000, Life Technologies, Carlsbad, CA, USA) |  | Alexa Fluor 488-labeled donkey anti-rabbit IgG antibodies (1:5000, Life Technologies, Carlsbad, CA, USA) |  | Alexa Fluor 488-labeled donkey anti-rabbit IgG antibodies (1:5000, Life Technologies, Carlsbad, CA, USA) |  | None |  | Alexa Fluor 488-labeled donkey anti-rabbit IgG antibodies (1:5000, Life Technologies, Carlsbad, CA, USA) |  | Alexa Fluor 488-labeled donkey anti-rabbit IgG antibodies (1:5000, Life Technologies, Carlsbad, CA, USA) |  | Alexa Fluor 488-labeled donkey anti-mouse IgG antibodies (1:5000, Life Technologies, Carlsbad, CA, USA) |  | None |  | Room temperature, 60 min |
| IHC: immunohistochemstry. IF: Immunofluorescence. WT: Western blotting. IBL: Immuno-Biological Laboratories. | | | | | | | | | | | | | | | | | | | | |
