# Supplementary material for: Podocyte Injury Caused by Indoxyl Sulfate, a Uremic Toxin and Aryl-Hydrocarbon Receptor Ligand
Source: PLoS One. 2014 Sep 22;9(9):e108448. doi: 10.1371/journal.pone.0108448 (PMC4171541; doi:10.1371/journal.pone.0108448)
Supplement: Table S2 — Primers used in this study. (DOCX) [file pone.0108448.s004.docx]

| **Species** | **Symbol** | **Gene name** | **ID** | **Forward (5'-3')** | **Reverse (5'-3')** | **Product size (bp)** |
| --- | --- | --- | --- | --- | --- | --- |
| Mouse | *18S rRNA* | 18S ribosomal RNA | NR_003278.3 | CGCCGGCGGCTTGGTGACTC | TTGCGCGCCTGCTGCCTTCC | 210 |
| Mouse | *Actb* | actin, beta | NM_007393 | ACTGCTCTGGCTCCTAGCAC | CAGCTCAGTAACAGTCCGCC | 196 |
| Mouse | *Actn4* | actinin alpha 4 | NM_021895 | TCCAGGACATCTCTGTGGAAG | CATTGTTTAGGTTGGTGACTGG | 216 |
| Mouse | *Ahr* | aryl-hydrocarbon receptor | NM_013464 | AGGCCAGGACCAGTGTAGAG | CCAGGTAATCTTGGATAGTGGAG | 149 |
| Mouse | *Aip* | aryl-hydrocarbon receptor-interacting protein | NM_016666 | ATCGCAAGACTTCGAGAGGAC | GAACTTCTTGCCAACGATGAG | 192 |
| Mouse | *Cd2ap* | CD2-associated protein | NM_009847 | CAAGATGCCTGGAAGACGA | GCACTTGAAGGTGTTGAAAGAG | 177 |
| Mouse | *Cyp1a1* | cytochrome P450, family 1, subfamily a, polypeptide 1 | NM_009992 | GAGCACTACAGGACATTTGAGAAG | ACCTTATCATCTGACAGCTGGAC | 119 |
| Mouse | *Il6* | interleukin 6 | NM_031168 | TGTATGAACAACGATGATGCAC | TGGTACTCCAGAAGACCAGAGG | 137 |
| Mouse | *Myh9* | myosin, heavy polypeptide 9, non-muscle | NM_022410 | AAGGACCAGGCTGACAAGG | GTCACGACAAATGGCAGGTC | 209 |
| Mouse | *Nphs1* | nephrosis 1 homolog, nephrin | NM_019459 | ACCTGTATGACGAGGTGGAGAG | TCGTGAAGAGTCTCACACCAG | 218 |
| Mouse | *Nphs2* | nephrosis 2 homolog, podocin | NM_130456 | AAGGTTGATCTCCGTCTCCAG | TTCCATGCGGTAGTAGCAGAC | 105 |
| Mouse | *Podxl* | podocalyxin-like | NM_013723 | TCCTAAGGCCGTGTATGAGC | GATGCCATGCAGACGATG | 153 |
| Mouse | *Synpo* | synaptopodin | NM_177340.2 | CATCGGACCTTCTTCCTGTG | TCGGAGTCTGTGGGTGAG | 90 |
| Mouse | *Tnfa* | tumor necrosis factor | NM_013693 | CGAGTGACAAGCCTGTAGCC | GAGAACCTGGGAGTAGACAAGG | 167 |
| Mouse | *Wt1* | Wilms tumor 1 homolog | NM_144783 | GGTATGAGAGTGAGAACCACACG | AGATGCTGACCGGACAAGAG | 137 |
